# Annotations

**Published:** 1899-10-21

**Authors:** 


### Annotations.

### Methods of Clinical Teaching.

Speaking at the Nottingham Medico-Chirurgical Society, Dr. Robert Saundby, among other interesting matters, discussed the question whether medical students ought to do any hospital work during their first and second years, and while on this subject he drew attention to the amount of time which is often wasted by students in and about hospitals, and to the lack of organisation which is too frequently visible in all that concerns the teaching of clinical medicine. Instead of the "going round" which is now the fashion, entering the wards and crowding round the beds, classes should be arranged for first and second years' men, which should take the form of demonstrations of clinical cases, the patients being brought into the room one by one, and the chief features and symptoms of their maladies being pointed out. This is a mode of teaching which, as Dr. Saundby says, is of the highest utility, and is far too little employed in this country. Among its great advantages are that such classes can be arranged to take place punctually; that there need be no loss of time over irrelevant matter, as of necessity occurs where a physician goes round the wards; and that the class can be much more easily kept in control than is the case in ward visits, where the men in the outer circle always have an excuse for inattention in the fact that they cannot see what is going on. Of course, we are quite aware that in many hospitals such classes are held, but they are not by any means as common as they might be. The ideal clinical lecture is that which is conducted in a well-lighted theatre, into the arena of which patients illustrating a particular disease or group of diseases are introduced, the case of each of them in turn being thoroughly described and commented upon. Such lectures, however, require careful preparation, and are apt to be shirked by teachers, who often prefer to substitute a systematic lecture, at which many of the students unfortunately go to sleep, or a ward visit at which many of them play. Hence clinical teaching suffers.

### Hospitals in Vienna.

In this month's *Nineteenth Century* there is an article by Miss C. O'Conor-Eccles describing the hospitals of Vienna, which is by no means complimentary to them. The brilliancy of Vienna as a medical school, and the facility with which graduates of all nationalities are able to obtain practical teaching in all the specialties, has perhaps led medical men who visit its hospitals to overlook, or at least to regard as mere customs of the country, many points in regard to which, so far as nursing and general comfort are concerned, patients in Austrian hospitals are distinctly badly of. The low class from which the nurses are recruited, the meagre salary, the "tip" system, which "seems little better than a system of blackmailing the patients," the sale of morning coffee to the patients by the nurses — which is one of their perquisites—the sale of beer to the patients from a canteen, the indifference to decency shown in examining the patient, who is "sounded, and punched, and questioned at all hours by successive bodies of students"—all these things and many more are related by Miss O'Conor-Eccles as illustrating the condition of the hospitals in Vienna, and especially of the great Allgemeines Krankenhaus. We fear that what she says is true, but perhaps it would be more correct to regard the condition of the hospitals as indicative of the low standard of comfort accepted as their due by the working classes in Austria, than to regard it as indicating ignorance of better things. Many things could be improved in Vienna besides the hospitals.

### Baths for the People.

A great deal has of late been done to increase the facilities for public bathing in the metropolis, and the process still goes on. Of the popularity of the swimming bath there seems to be no question, and as a healthy amusement swimming is highly to be recommended. One cannot but feel, however, that in the planning of some of these great public baths an undue amount of attention is being paid to swimming as an athletic exercise, and that bathing as a means to cleanliness is being placed rather in the background. With the steady extension of the system of living in tenements rather than in self-contained houses comes an increasing difficulty in obtaining that bathing accommodation which, to those engaged in manual labour, is one of the first essentials to health, and a sanitary authority which affords cheap and readily-accessible arrangements for the cleansing of the person deserves well of its constituents. For this purpose the swimming bath is of no use, in fact, no one ought to be admitted to it until he has thoroughly cleansed himself, and we venture to doubt whether the slipper bath, which is the usual alternative, is the best arrangement which could be devised. A report has recently been presented to the Liverpool Corporation, by the engineer and superintendent of the Municipal Baths in that city, in which the erection of baths of the Lassar-Grove type is strongly advocated. Such baths exist in considerable numbers in Germany, and there are a few in England, but not by any means as many as there might be. The form of bath in question is a spray or rain bath, at any desired temperature, terminating, if it is thought fit, with a cold shower. Among the economical advantages of this kind of bath are a smaller space required, the smaller quantity of water used, and the shorter time occupied in the process. To the bather, however, the great and preponderating advantage is that the bath is clean. The slipper bath, even when used by cleanly people, is not an entirely attractive arrangement unless very considerable attention be paid to the cleansing of the bath itself; and when baths of this kind are used by dirty people—used, that is, for the purpose of removing dirt—they are apt to become definitely repulsive unless much labour is expended in cleaning them after each time of use; and labour is expensive and often inefficient. In the rain bath, on the contrary, the bather, except by the soles of his feet, touches nothing which has been touched by anyone else. He stands in a flowing rain of clean water, and so soon as he departs a single pull at the handle makes all ready for the next comer. There can be no doubt that the rain bath is the proper form of bath for cleansing purposes. Liverpool has decided to erect a set on the principle we have just described, and it is to be hoped other towns will do the same.

